# Efflux Activity Differentially Modulates the Levels of Isoniazid and Rifampicin Resistance among Multidrug Resistant and Monoresistant *Mycobacterium tuberculosis* Strains

**DOI:** 10.3390/antibiotics7010018

**Published:** 2018-03-03

**Authors:** Diana Machado, João Perdigão, Isabel Portugal, Marco Pieroni, Pedro A. Silva, Isabel Couto, Miguel Viveiros

**Affiliations:** 1 Global Health and Tropical Medicine, GHTM, Instituto de Higiene e Medicina Tropical, IHMT, Universidade NOVA de Lisboa, UNL, Lisboa 1349-008, Portugal; diana@ihmt.unl.pt (D.M.); icouto@ihmt.unl.pt (I.C.); 2 iMed.ULisboa, Instituto de Investigação do Medicamento, Faculdade de Farmácia, Universidade de Lisboa, Lisboa 1649-003, Portugal; jperdigao@ff.ulisboa.pt (J.P.); iportugal@ff.ulisboa.pt (I.P.); 3 P4T Group, University of Parma, Parco Area delle Scienze 27/A, Parma 43124, Italy; marco.pieroni@unipr.it; 4 Department of Food and Drug, University of Parma, Parco Area delle Scienze 27/A, Parma 43124, Italy; 5 Núcleo de Pesquisa em Microbiologia Médica (NUPEMM), Faculdade de Medicina, Universidade Federal do Rio Grande, Rio Grande, Porto Alegre 96200-190, Brazil; pedrefurg@gmail.com

**Keywords:** efflux inhibitors, lineages, mutations, resistance levels, tuberculosis, verapamil

## Abstract

With the growing body of knowledge on the contribution of efflux activity to *Mycobacterium tuberculosis* drug resistance, increased attention has been given to the use of efflux inhibitors as adjuvants of tuberculosis therapy. Here, we investigated how efflux activity modulates the levels of efflux between monoresistant and multi- and extensively drug resistant (M/XDR) *M. tuberculosis* clinical isolates. The strains were characterized by antibiotic susceptibility testing in the presence/absence of efflux inhibitors, molecular typing, and genetic analysis of drug-resistance-associated genes. Efflux activity was quantified by real-time fluorometry. The results demonstrated that all the *M. tuberculosis* clinical strains, susceptible or resistant, presented a faster, rapid, and non-specific efflux-mediated short-term response to drugs. The synergism assays demonstrated that the efflux inhibitors were more effective in reducing the resistance levels in the M/XDR strains than in the monoresistant strains. This indicated that M/XDR strains presented a more prolonged response to drugs mediated by efflux compared to the monoresistant strains, but both maintain it as a long-term stress response. This work shows that efflux activity modulates the levels of drug resistance between monoresistant and M/XDR *M. tuberculosis* clinical strains, allowing the bacteria to survive in the presence of noxious compounds.

## 1. Introduction

Tuberculosis (TB) is a major public health problem worldwide, accounting for an estimated 10.4 million new cases and 1.3 million deaths in 2016 [1]. Despite worldwide efforts to reduce the global burden of tuberculosis, multidrug resistant (MDR) and extensively drug resistant (XDR) tuberculosis (continue to increase, leaving very limited therapeutic options for these patients. The emergence of MDR (simultaneous resistance to isoniazid and rifampicin) and XDR (MDR with additional resistance to second-line injectables and fluoroquinolones) *Mycobacterium tuberculosis* has become a major public health concern worldwide. There was an estimate of 490,000 new MDRTB cases with approximately 200,000 deaths in 2016. Among these, 6.2% were anticipated to be XDRTB cases [1].

*M. tuberculosis* develops drug resistance mainly by mutations in genes that code for the drug targets [2], the impermeability of its cell wall, and the activity of efflux pumps [3,4,5,6,7,8]. Resistance mediated by efflux has been described as an important contributor to drug resistance in several bacterial pathogens [9]. Efflux pumps are transmembrane proteins involved in the extrusion of noxious compounds and cellular metabolites from the cells into the external environment, using cellular energy derived from ATP (Adenosine triphosphate) or the proton motive force [10]. They are associated with the transport of a wide range of structurally unrelated antimicrobials, preventing them from reaching their targets and being responsible for the development of MDR phenotypes [11]. These MDR phenotypes are dependent of the constitutive or inducible expression of their efflux systems [3,11,12] where the antibiotics act like inducers through the regulation of the expression of efflux pumps [13].

The role of efflux mechanisms in drug resistance in *M. tuberculosis* has been demonstrated over the last years [6,7,8,14,15]. Some of these putative efflux pumps have been associated with the transport of fluoroquinolones, isoniazid, rifampicin, ethambutol, β-lactams, doxorubicin, aminoglycosides, macrolides, tetracycline, and dyes, among others. Of note is that the resistance by efflux was already described as being involved in the *M. tuberculosis* resistance to bedaquiline, the most recent drug approved for the treatment of MDRTB [16]. Our previous works showed that the efflux pumps Mmr, MmpL7, Rv1258c, P55, Rv1218c-Rv1217c, Rv2459, and EfpA are overexpressed in the presence of antibiotics, demonstrating the contribution of these pumps to a genotype-independent resistance phenotype [17,18]. The stress imposed by a subinhibitory antibiotic concentration results in an increased efflux activity, allowing the selection of spontaneous mutants with clinically significant resistance levels [7,19,20].

The demonstration of the involvement of efflux pumps on the emergence of *M. tuberculosis* drug resistance makes these proteins interesting targets for the discovery of novel drugs. Because efflux is an important contributor to drug resistance, the identification and characterization of mycobacterial efflux inhibitors is an approach for the development of new effective antituberculosis therapies. Efflux inhibitors have been shown to potentiate the activity of several antituberculosis drugs. Compounds like thioridazine and verapamil have efflux inhibitory properties and inhibit the in vitro and ex vivo growth of *M. tuberculosis* strains alone or in combination with antimycobacterial drugs [17,19,21,22,23]. Thioridazine has demonstrated significant activity against MDRTB in a murine model of infection [24] and it has been successfully employed to treat XDRTB patients on the basis of compassionate reasons [25]. Verapamil has been shown to be the most potent mycobacterial efflux inhibitor to date, being able to enhance the inhibitory activity of isoniazid [19] and rifampicin [26] in *M. tuberculosis* clinical strains. Recently, it was demonstrated that efflux inhibition promoted by verapamil can potentiate the activity of bedaquiline [27]. It was also showed that the addition of verapamil accelerates the bactericidal and sterilising activities of tuberculosis therapy in a mouse model [28]. Beyond their antimycobacterial activity, these compounds also present immunomodulatory abilities on *M. tuberculosis*-infected macrophages and efflux inhibitors can enhance the killing of *M. tuberculosis* by macrophages [17,29,30]. Further evidence in favour of the usefulness of these compounds is reported in the studies by Adams et al. [22,23], wherein the selective pressure exerted by the macrophage on internalized *M. tuberculosis*-induced bacterial efflux pumps and thereby drug-tolerance. Additionally, these authors also showed that the macrophage drug-induced tolerance can be reduced by verapamil or its metabolites. Despite this, little is known about how the efflux activity influences the resistance levels among different *M. tuberculosis* lineages and different drug resistant mutational backgrounds.

Previously, we showed that the drug resistant phenotypes in *M. tuberculosis* are a combination of drug efflux and the presence of target-gene mutations, and that the efflux activity modulates the levels of antibiotic resistance by complementing the resistance due to the presence of target-gene mutations [18]. Our results demonstrated the existence of a broad-spectrum synergistic interaction between antibiotics and efflux inhibitors. However, we also noted that the efflux inhibitors were more active in reducing the minimum inhibitory concentration (MIC) values of isoniazid, rifampicin, or both in MDR and XDR *M. tuberculosis* strains overexpressing efflux pumps than in the monoresistant strains [18]. This data seems to indicate that the efflux activity may be strain-dependent and the efflux inhibitors exert their greatest effect on strains with increased efflux activity. The purpose of the present work is to study the correlation between the effect of efflux inhibitors and the genetic background, drug resistance profile and efflux activity in clinical strains of *M. tuberculosis* in order to describe how the efflux activity modulates the levels of resistance in strains presenting different drug-resistance associated mutations and genetic backgrounds. Understanding the dynamic underlying bacterial antibiotic-induced systems could provide a route for new chemotherapeutic approaches.

## 2. Results

### 2.1. Characterization of the M. tuberculosis Strains According to Their Efflux Capacity

The 18 *M. tuberculosis* strains were firstly characterized according to their efflux capacity using a fluorometric method that detects in real-time the accumulation and efflux of ethidium bromide in bacterial cells loaded with this fluorophore [19,31,32]. For this purpose, we selected a panel of susceptible, monoresistant, and M/XDR *M. tuberculosis* clinical isolates. These strains were chosen in order to include the most prevalent mutations associated with resistance to isoniazid and rifampicin. As controls, we selected three pan-susceptible *M. tuberculosis* strains and the H37Rv reference strain (Table 1).

**Figure 1:**
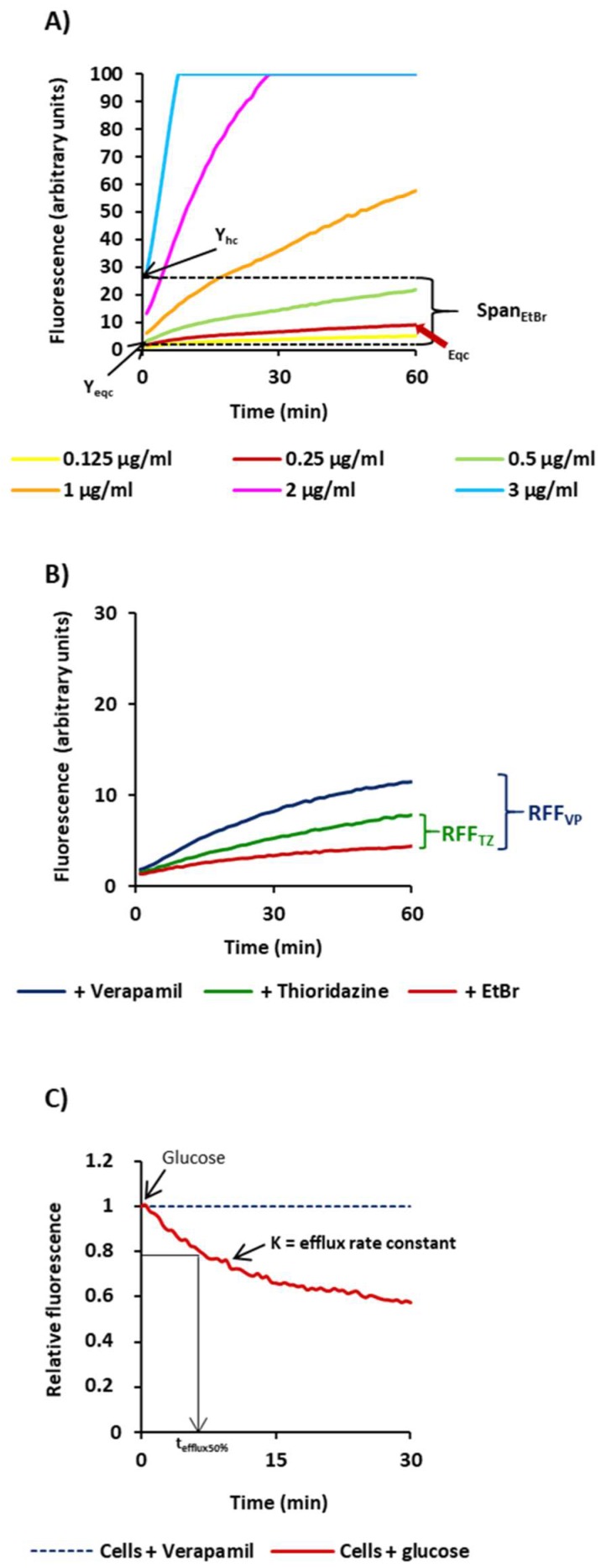
Schematic representation of parameters used for the characterization of the efflux activity of the *M. tuberculosis* strains. Presented in the Figure are the results obtained for the H37Rv strain. (**A**) SpanEtBr corresponds to the difference between the ethidium bromide fluorescence values at t_0_ of the highest concentration tested (Y_hc_) and the fluorescence value at t_0_ of the equilibrium concentration of ethidium bromide (Y_eqc_); (**B**) RFF (relative final fluorescence) is a measure of how effective a compound is on the inhibition of ethidium bromide efflux (at a given concentration) by comparison of the final fluorescence at the last time point (60 min) of the treated cells with the cells in presence of ethidium bromide only [19,32]; (**C**) efflux rate constant, or K value, and the t_efflux50%_ that corresponds to the time required for the cells to extrude half of the preloaded dye; the efflux of ethidium bromide is initiated at t_0_ by the addition of 0.4% glucose and is 50% complete at t_efflux50%_.

Figure 1 illustrates the results of the ethidium bromide fluorometric assays and the parameters used to characterize the efflux capacity of the 18 *M. tuberculosis* strains, giving as example the results obtained for strain H37Rv.

Initially we determined the Span_EtBr_ index as a measure of the capacity of each strain to accumulate ethidium bromide. This value corresponds to the difference between the ethidium bromide fluorescence values at t_0_ of the highest concentration tested (Y_hc_) and the fluorescence value at t_0_ of the equilibrium concentration of ethidium bromide (Y_eqc_) (Figure 1). High Span_EtBr_ indexes indicated increased capacity to accumulate ethidium bromide. The equilibrium concentration is the concentration where the uptake of ethidium bromide by the cell equals its efflux and corresponds to the first concentration that gives no more than 10 units of fluorescence at the end of the 60 min of the accumulation assay (indicated by the red arrow on the graph). The results obtained for the strains tested are shown in Table 2.

The accumulation of ethidium bromide for the *M. tuberculosis* strains took place at concentrations above 0.5 µg/mL for Mtb#3, Mtb#8, Mtb#11, and Mtb#12; 0.25 µg/mL for H37Rv, Mtb#1, Mtb#4, Mtb#6, Mtb#7, Mtb#13, Mtb#14, Mtb#16, and Mtb#17; and 0.125 µg/mL for Mtb#2, Mtb#5, and Mtb#9. Analysing the data presented in the Table 2, we can observe that the susceptible strains presented higher Span_EtBr_ values than the drug resistant strains, which is indicative of an increased accumulation of ethidium bromide at higher concentrations (lesser ability to efflux). Concerning the drug resistant strains, there was no significant difference on the Span_EtBr_ values between the monoresistant strains and the M/XDR strains (Table 2). These results showed that the drug resistant strains (monoresistant and M/XDR) can handle higher concentrations of ethidium bromide than the susceptible ones having a higher efflux capacity.

**Figure 2:**
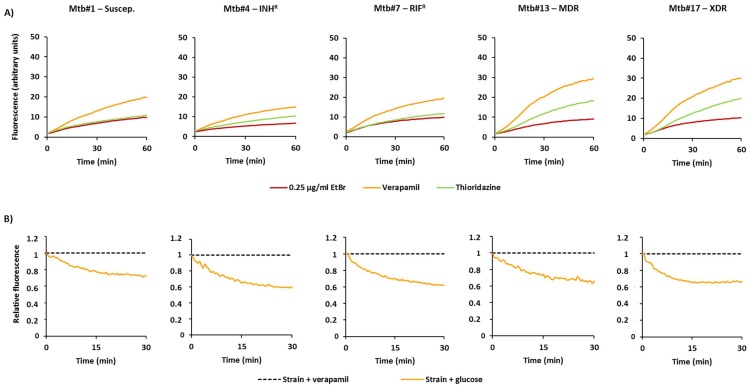
Accumulation and efflux of ethidium bromide of the *M. tuberculosis* strains. (**A**) Accumulation of ethidium bromide in the presence of efflux inhibitors. In these cases, the strains were loaded with 0.25 µg/mL of ethidium bromide in the presence of verapamil or thioridazine at ½ MIC; (**B**) Efflux of ethidium bromide. Strains were loaded with ethidium bromide at 0.25 µg/mL; efflux took place in the presence of glucose which was inhibited by verapamil at ½ MIC.

Next, we compared the rate of accumulation of ethidium bromide in the presence of the efflux inhibitors as an indirect method to evaluate the efflux capacity of these strains (Table 2; Figure 2A). For this purpose, we selected verapamil and thioridazine, two well-known efflux inhibitors of *M. tuberculosis* efflux systems [7,17,19,22,23,33], and determined the RFF indexes. The RFF index is measure of how effective a compound is on the inhibition of ethidium bromide efflux (at a given concentration) by comparison of the final fluorescence at the last time point (60 min) of the treated cells with the cells in presence of only ethidium bromide. At the same time, high levels of inhibition, or high RFF values, indicate higher efflux activity of the strain and vice versa. As showed in Table 2, verapamil promoted the highest levels of accumulation of ethidium bromide (RFF > 1) in 14 out of 16 of the strains tested. Thioridazine showed to be less potent in the inhibition of ethidium bromide efflux than verapamil as previously described [17,33]. These results indicated that all strains have active efflux systems that can be inhibited by verapamil and, in less extent, by thioridazine.

Finally, to directly compare the efflux capacity of each strain, we further determined the efflux rate constant, or K value, and the t_efflux50%_ that corresponds to the time required for the cells to extrude half of the preloaded dye (Table 2). To preload the cells with ethidium bromide, we selected verapamil. Shown in Figure 2B are the results for selected strains on each of the drug resistance categories. The efflux rate constants obtained for the 14 strains, whose efflux activity could be inhibited by verapamil at high levels (RFF > 1), varied between 0.07 and 0.14. For the two strains with reduced efflux activity, Mtb#3 and Mtb#12, the efflux rate constants were 0.06 and 0.05, respectively.

Concordantly, these strains were the ones that showed the slowest efflux rates as they took more time to extrude half of the preloaded dye; strain Mtb#3 took 11.98 min and Mtb#12 took 14.06 min. On the other hand, the t_efflux50%_ of the remaining strains was between 4.39 min and 8.95 min, where Mtb#8 had the lowest t_efflux50%_ and Mtb#4 had the higher t_efflux50%_. No correlation could be observed between the different drug susceptibility profiles of the strains tested and the effect of both compounds on the ethidium bromide efflux inhibition confirming the results obtained on the ethidium bromide accumulation assays.

Overall, the results showed that (i) all strains presented efflux activity as a short-term stress response to drugs, and (ii) the drug resistant strains presented increased capacity to handle higher concentrations of toxic compounds, in this case, ethidium bromide, when compared with the susceptible strains, revealing an enhanced efflux capacity independently of their resistance phenotype.

### 2.2. Effect of Efflux Inhibitors on the Resistance Levels to Rifampicin and Isoniazid

The MICs of the antibiotics and the efflux inhibitors for the *M. tuberculosis* strains included in this study are summarized in Table 1. All the strains carried mutations in *katG* or *inhA* genes that have been described to be involved in the resistance to isoniazid, mutations in the *rpoB* gene which are associated with resistance to rifampicin, or both. The MICs of isoniazid for the strains presenting the mutation S315T in *katG* are between 10 and 20 µg/mL contrasting with the MICs presented by strains with mutations in *inhA* promotor (0.4 µg/mL) or *inhA* promotor plus ORF (Open Reading Frame) mutations (3 µg/mL). Regarding rifampicin, the strains with mutations in *rpoB* presented MICs between 20 µg/mL and 640 µg/mL. The MICs of both efflux inhibitors remained practically unchanged among all strains (one dilution noted).

Subsequently, the effect of verapamil and thioridazine on the susceptibility levels of these strains was evaluated (Table 3). Verapamil increased the susceptibility to isoniazid in 8/11 strains and to rifampicin in 8/13 strains. In both cases, all the strains were at least MDR. Thioridazine showed a weaker effect, increasing the susceptibility to isoniazid in 5/11 strains and to rifampicin in 3/13 strains, all of them MDR. Comparing the data gathered for the monoresistant strains with the data of the MDR strains, it was noticed that verapamil and thioridazine were more effective in reducing the resistance levels of the M/XDR strains than of the monoresistant strains. This data indicated that the M/XDR strains presented a more prolonged response to drugs mediated by efflux as observed by the effect of verapamil on the susceptibility levels of isoniazid and rifampicin of these strains.

Nevertheless, although the resistance levels could not be reduced in the monoresistant strains, a synergistic effect was observed. The inhibition of growth of the monoresistant strains in presence of isoniazid or rifampicin in combination with the inhibitors showed a delay of 1 to 3 days between the tube containing the antibiotic plus the efflux inhibitor and the tube containing only the antibiotic (data not shown). The synergistic effect depended on the drug combination and the concentration of antibiotics tested, and demonstrates the existence of efflux activity in these strains but at lower levels compared to the M/XDR strains.

Analysing the data collected in Table 1, Table 2 and Table 3, no correlation could be found between the genotype and the efflux activity of these strains.

## 3. Discussion

With the growing body of knowledge about the contribution of efflux activity to *M. tuberculosis* drug resistance, increased attention has been given to the use of efflux inhibitors as adjuvants of tuberculosis therapy [7,8,34]. In previous works, we showed that *M. tuberculosis* drug resistance levels are a balance between the presence of a mutation in the genes coding the drug target and active efflux [18]. We observed that strains presenting the same mutation conferring antibiotic resistance had different MICs and that the different resistance levels found could be reduced by efflux inhibitors. Although full susceptibility was not restored, the existence of a broad-spectrum synergistic interaction between antibiotics and efflux inhibitors was shown. However, we also noticed that the efflux inhibitors tested were significantly more effective against the M/XDR strains once compared with their monoresistant counterparts. An evident question posed by these results is why the efflux inhibitors are more effective on M/XDR strains. We hypothesized that the efficacy of the efflux inhibitors could be strain-dependent, i.e., it may depend on the (i) genetic background or (ii) type of mutation in the genes associated with resistance. To answer this question, in this study we investigated the effect of the efflux inhibitors verapamil and thioridazine on the efflux levels of a panel of *M. tuberculosis* clinical strains susceptible, isoniazid and rifampicin monoresistant, and M/XDR*,* in order to describe how the efflux activity modulates the levels of resistance in strains presenting different phylogenetic lineages and drug-resistance associated mutations.

The strains selected were the *M. tuberculosis* H37Rv, a worldwide used laboratory reference strain fully susceptible to the first- and second-line antibiotics, and a panel of 17 clinical strains differing on their phylogenetic origins, drug susceptibilities, and drug resistance associated mutations. Firstly, we characterized all the *M. tuberculosis* strains according to their efflux capacity by real-time fluorometry to analyse the short-term efflux-mediated responses to the presence of toxic compounds measuring the cells response via efflux of ethidium bromide. With this method, we detected and measured the efflux activity in all strains. This activity could be inhibited in the presence of verapamil, and less efficiently by thioridazine, regardless of the phenotype or genotype of the strain in study. The efflux assays clearly demonstrated that all the clinical strains, susceptible or resistant, presented a faster, very rapid, and non-specific efflux-mediated short-term response, when compared with the drug-naïve laboratory strain H37Rv, allowing the cells to cope in the presence of toxic compounds, as previous seen on laboratory efflux-induced strains [19].

Concomitantly, the determination of the MICs of the antibiotics in the presence of the efflux inhibitors provided information regarding the efflux-mediated long-term response of the cells to the presence of noxious compounds, like ethidium bromide or antibiotics. Verapamil and thioridazine were able to reduce the resistance levels of isoniazid, rifampicin, or both in the M/XDR strains, and in particular it was possible to reverse the resistance to rifampicin in one of the MDR strains (Mtb#10). Interestingly, the same kind of results were not observed for the monoresistant strains. No reduction of the MICs in the presence of the efflux inhibitors was observed for the rifampicin or isoniazid monoresistant strains. Despite the absence of a reduction in the antibiotic resistance levels in the presence of the efflux inhibitors, a synergic effect between the efflux inhibitors and the antibiotics was noted through the evaluation of the delay in the time to detection (TTD) of growth, demonstrating some positive drug interaction, as expected by the presence of active efflux systems in these strains, as we have highlighted before. Efflux activity in the monoresistant strains occurs at lower levels compared to the M/XDR strains but both maintain it as a long-term stress response to drugs. Clearly, in the M/XDR strains this activity is increased, contributes to an overall high-level resistance to isoniazid and rifampicin, and can be reduced by the efflux inhibitors. These findings provide further evidence on the role of efflux as a global stress response in *M. tuberculosis* and corroborate the previous notion that *M. tuberculosis* clinical strains are primed to efflux toxic compounds [19].

Moreover, the data obtained showed that the efflux activity and the effect of the efflux inhibitors does not depend on the strain genetic background and mutation(s) associated with drug resistance. The rifampicin resistance levels were reduced from 320 µg/mL to 20 µg/mL in four M/XDR strains harbouring the S531L *rpoB* mutation but not in the two rifampicin monoresistant strains presenting the same mutation. Also, the rifampicin resistance of the MDR strain Mtb#10, presenting the mutation H526Y on *rpoB*, was reversed from 20 µg/mL to 1 µg/mL in the presence of verapamil and thioridazine, but not in the rifampicin monoresistant strain (Mtb#6) that carried the same mutation. The same results were observed for the C-15T mutation on the *inhA* promoter between the M/XDR strains and the isoniazid monoresistant strain presenting the same mutation. Overall, these results are in concordance with those obtained with the real-time fluorometry assays (evaluation of the short-term efflux response), where it was shown that the efflux activity and the effect of the efflux inhibitors on this activity is independent on the drug-resistant associated mutations of the strains, but with the M/XDR strains recording higher levels of efflux activity.

Mutations such as the S315T in *katG*, C-15T in *inhA* promoter, and S531L in *rpoB* are predominant in *M. tuberculosis* clinical isolates and have been associated with successful transmission [35,36,37]. These mutations are the most frequently found in clinical isolates [38], meaning that the *M. tuberculosis* population within the host may be undergoing adaptive evolution to improve bacterial fitness and efflux contribute for their survival and epidemiological success.

## 4. Materials and Methods

### 4.1. M. tuberculosis Strains

The strains studied are described in Table 1. These strains are part of the culture collection of the Grupo de Micobactérias, Unidade de Microbiologia Médica, of the Instituto de Higiene e Medicina Tropical (IHMT), Universidade NOVA de Lisboa (UNL). The panel of *M. tuberculosis* strains studied was selected according to their susceptibility pattern and the presence of the most common mutations associated with resistance to isoniazid and rifampicin in order to include susceptible, isoniazid monoresistant, rifampicin monoresistant, MDR, and XDR. Informed consent was not required for this study since it is a retrospective study from which all patient identification was unlinked from the results, and no patient information was collected. *M. tuberculosis* H37Rv ATCC27294^T^ was obtained from the American Type Culture Collection (Manassas, VA, USA) and was included as control strain.

### 4.2. Antimicrobials and Reagents

The antibiotics isoniazid and rifampicin, the efflux inhibitors verapamil and thioridazine, the efflux substrate ethidium bromide (EtBr), Tween 80, phosphate buffered saline (PBS), dimethyl sulfoxide (DMSO), and glucose, were obtained from Sigma-Aldrich (St. Louis, MO, USA). Rifampicin was prepared in DMSO, while the remaining drugs were prepared in sterile deionized water. The stock solutions were aliquoted and stored at −20 °C and the working solutions freshly prepared on the day of the experiment. The lyophilized drugs (BACTEC MGIT 960 SIRE and PZA kits; SIRE, streptomycin, isoniazid, rifampicin, ethambutol; PZA, pyrazinamide) used in the standard susceptibility testing were purchased from Becton Dickinson (Diagnostic Systems, Sparks, MD, USA) and the stock solutions prepared as per the manufacturer’s instructions. BD Difco Middlebrook 7H9, BD BBL OADC (oleic acid/albumin/dextrose/catalase) supplement and the Mycobacteria Growth Indicator Tubes (MGITs) were from Becton Dickinson.

### 4.3. Susceptibility Testing

#### 4.3.1. Growth of the Strains

For first-line susceptibility testing, MIC determination, and synergism assays, the *M. tuberculosis* strains were inoculated in MGITs tubes supplemented with 10% OADC and incubated at 37 °C in the BACTEC MGIT 960 (MGIT 960) system until they reached 100–200 growth units (GUs) and were used directly for testing. Cultures with 200 < GU < 2000 were diluted to 100–200 GU with a sterile saline solution and used for testing. Growth of the cultures was monitored with the Epicenter V5.80A software equipped with the TB eXIST module (Becton Dickinson).

#### 4.3.2. First-Line Drug Susceptibility Testing

The MGIT 960 was used for first-line drug susceptibility testing according to the manufacturer’s instructions. The MGIT tubes were inoculated with 0.8 mL of OADC, 0.1 mL of the antibiotic at the critical concentrations (0.1 μg/mL for isoniazid, 1 μg/mL for rifampicin, 1 μg/mL for streptomycin, 5 μg/mL for ethambutol, 100 μg/mL for pyrazinamide) and 0.5 mL of the suspension of the strain. For preparation of the drug-free proportional control, the strain suspension was diluted 1:100 (1:10 for PZA) with a sterile saline solution and 0.5 mL inoculated in the tube. The results were interpreted as follows: if the GU of the tubes containing the drug were >100 when the proportional control reached 400 GU, they were considered to be resistant to the respective concentration. If the GU of the tube containing the drug was <100, they were considered susceptible [39].

#### 4.3.3. MIC Determination of Antibiotics and Efflux Inhibitors

MIC determination of the antibiotics and efflux inhibitors was performed within the MGIT 960 and the Epicenter V5.80A/TB eXIST software. The inoculum of the strains and the MGIT tubes were prepared as described above. At the time of testing, two-fold serial dilutions were prepared to achieve the desired concentrations and 0.1 mL added to the corresponding drug-containing tubes. For the proportion testing it was prepared two drug-free controls: the absolute control (1:1) and the proportional control (1:100). The MIC was considered as the lowest concentration with GU < 100 when the drug-free proportional control tube reached a GU value of 400.

#### 4.3.4. Quantitative Drug Susceptibility Testing of Antibiotics in Presence and Absence of Inhibitors

Quantitative drug susceptibility testing of rifampicin and isoniazid conducted using the MGIT 960 and the Epicenter V5.80A/TB eXIST. Isoniazid was tested at 0.1, 1, 3, and 10 µg/mL and rifampicin at 1, 4, and 20 µg/mL. For the quantification of the antibiotic resistance levels of each strain, the results were interpreted as per Cambau et al. [38] as follows: isoniazid low-level resistance when resistant (R) at 0.1 and susceptible (S) at 1 µg/mL; isoniazid high-level resistance when R ≥ 1; rifampicin low-level resistance when R at 4 and S at 20 µg/mL; rifampicin high-level resistance when R ≥ 20 µg/mL. The preparation of the drug containing tubes and controls was done as described above. For the susceptibility testing for the antibiotics in the presence of the inhibitors, the tubes containing 0.1 mL of the antibiotics at the desired concentrations were inoculated with 0.1 mL of the inhibitor to a final concentration of 1/2 or 1/4 their MIC, depending on the strain. These concentrations were selected in order to not compromise the growth of the strains following the protocol described above. The resistance levels for each combination (antibiotic + efflux inhibitor) were quantified as describe above.

### 4.4. Ethidium Bromide Real-Time Fluorometry

To detect the accumulation and extrusion of ethidium bromide, a common substrate of several efflux pumps, we applied a real-time fluorometric method using a Rotor-Gene 3000 thermocycler (Corbett Research, Sydney, Australia) [31,40]. The assays were performed as previously described [19]. The strains were grown in 10 mL of MB7H9 medium, 10% OADC, and 0.05% Tween 80, at 37 °C, without stirring, until they reached an approximate OD_600_ of 0.8. The cells were collected by centrifugation at 2940× *g*, for 3 min at room temperature. The supernatant was discarded, the pellet washed, resuspended in PBS, and centrifuged as before. This procedure was performed twice.

For the ethidium bromide accumulation assays, the washed cells were re-suspended in PBS and the OD_600_ adjusted to 0.8. To determine the equilibrium concentration, i.e., the lowest concentration of ethidium bromide that causes accumulation, 50 µL of the cell suspension was added to 0.2 mL tubes containing different concentrations of ethidium bromide that ranged from 0.125 to 3 µg/mL. The final OD_600_ of the bacterial suspension in the assay was 0.4. The assays were conducted at 37 °C in a Rotor-Gene 3000, and the fluorescence of ethidium bromide was measured (530/585 nm) at the end of each cycle of 60 s, for 60 min. After this, the effect of the efflux inhibitors verapamil and thioridazine on the accumulation of ethidium bromide was evaluated for each strain. These assays were performed like described above with each efflux inhibitor at 1/2 or 1/4 of the MIC and ethidium bromide at the higher concentration that does not cause accumulation determined for each strain. To quantify the effect of the inhibitors in the accumulation of ethidium bromide, for each strain we determined the relative final fluorescence (RFF) as previously described [41]. RFF values above 0 indicated that cells accumulate more ethidium bromide under the condition used than the non-treated cells. Each assay was performed in triplicate and the results presented correspond to the average of three independent assays (±SD).

For the efflux assays, the washed cells were resuspended in PBS and the OD_600_ adjusted to 0.4. The ethidium bromide loaded cells were prepared by incubating the cell suspension in the presence of the highest concentration of ethidium bromide that does not cause accumulation and verapamil at 1/2 (or 1/4) of the MIC at room temperature during 60 min. After EtBr accumulation, the cells were collected by centrifugation at 2940× *g* during 3 min, room temperature, and resuspended in PBS to an OD_600nm_ of 0.8. Then, 50 µL of the suspension was added to 0.2 mL tubes containing (1) PBS only; (2) PBS plus glucose, to a final concentration of 0.4%; (3) verapamil only; and (4) glucose to a final concentration of 0.4% plus verapamil. The final OD_600_ of the bacterial suspension in the assay was 0.4. The fluorescence of ethidium bromide was measured, as described above. The fluorescence was acquired first, at the end of 15 s and at the end of every 30 s during the following 30 min. The efflux activity was quantified by comparing the fluorescence data obtained for the cells under conditions that allow maximum efflux (incubation at 37 °C in the presence of glucose and absence of a compound) against the data from the control tube that contains the ethidium-bromide-loaded cells under conditions that inhibit the efflux (with a compound and absence of glucose). The normalized data (Y, fluorescence vs. X, time) was plotted using GraphPad Prism (GraphPad Software, La Jolla, CA, USA) [42]. Ethidium bromide efflux curves were fitted using a single exponential decay equation as follows: Y=(Y0−Plateau)∗exp(−K∗X)+Plateau, where X is the time, Y is the ethidium bromide efflux that starts at Y_0_ and decays to the plateau in one phase; the plateau is Y at infinite times. The plateau has the same units as Y and the K or slope is the efflux rate constant.

### 4.5. Genotypic Characterization of the Strains

(i) DNA extraction: Genomic DNA was extracted using the QIAamp DNA mini kit (QIAGEN, GmbH, Hilden, Germany) according to the manufacturer’s instructions.
(ii) Screening of mutations: The most common mutations in *rpoB*, *katG*, and the *inhA* promoter were screened using the system Genotype MTBDR*plus* V2 (Hain Lifescience GmbH, Nehren, Germany) according to the manufacturer’s instructions.
(iii) DNA sequencing: The analysis of internal fragments of the genes *katG*, *inhA*, and *rpoB* was performed according to Machado et al. [43].
(iv) Strain typing: Spoligotyping was performed as previously described [44]. Detection of the hybridization patterns was carried out using the ECL Chemiluminescence Detection System (GE Healthcare, Cleveland, OH, USA).

### 4.6. Statistical Analysis

Statistical analysis was carried out using the Student’s *t*-test. A * *p* value < 0.05 was considered statistically significant and highly significant when ** *p* < 0.01 and *** *p* < 0.001 (two-tailed test).

## 5. Conclusions 

The acquisition of drug resistance by the *M. tuberculosis* strains is frequently associated with a decreased in the bacterial fitness and reduced transmissibility. Nevertheless, *M. tuberculosis* can evolve and adapt to reduce the biological cost of MDR by the acquisition of compensatory mechanisms to ameliorate the cost of its resistance, allowing its survival and spread in the population. The findings gathered in this study emphasize the contribution of efflux activity in *M. tuberculosis* towards adaptive evolution. The overactivity of efflux pumps should be considered when studding the impact of MDR in the transmission dynamics and virulence of *M. tuberculosis* clinical strains. Drug resistance mediated by efflux has become an important issue since it helps the bacteria to survive in the presence of antimicrobial compounds. In this sense, the potential use of efflux inhibitors in combination with antibiotics may be valuable as adjunct for a combined therapeutic approach. The overactivity of efflux pumps can influence the bacterial dynamics within the population in the presence of antibiotic pressure, until mutations in the drug targets arise and establish in the population [19]. The final resistance level is a combination between the efflux of the drug and the presence of the mutations [18]. This work complemented our previous findings now demonstrating how efflux activity modulates the levels of drug resistance between monoresistant and M/XDR *M. tuberculosis* clinical strains allowing the bacteria to survive for longer periods in the presence of noxious compounds.

Further studies into the mechanisms of drug resistance that alleviate the biological cost of drug resistance will help to better understand the emergence, epidemiological success, and dissemination of antimicrobial resistance in the clinical setting and also aid in its management and prevention.

## Figures and Tables

**Table 1:** Spoligotype family, drug susceptibility profile and mutational background of the *M. tuberculosis* strains.

Strain	Spoligotype SIT/Clade	AST	Drug Resistance Mutational Background		MICs (µg/mL)			
					Antibiotics		Efflux Inhibitors	
			INH	RIF	INH	RIF	VP	TZ
H37Rv	H37Rv	Suscep.	wt	wt	0.1	1	256	15
Mtb1	LAM9—Orphan	Suscep.	wt	wt	0.1	1	256	30
Mtb2	Unknown—SIT105	Suscep.	wt	wt	0.1	1	256	15
Mtb3	T1—SIT612	INH^R^	*katG* S315T	wt	10	1	256	15
Mtb4	LAM1—SIT20	INH^R^	*inhA* C-15T	wt	0.4	1	256	15
Mtb5	LAM2—SIT53	RIF^R^	wt	*rpoB* H526D	0.1	320	256	15
Mtb6	Beijing—SIT1	RIF^R^	wt	*rpoB* H526Y	0.1	320	128	15
Mtb7	LAM1—SIT2576	RIF^R^	wt	*rpoB* S531L	0.1	320	256	15
Mtb8	LAM1—SIT2576	RIF^R^	wt	*rpoB* S531L	0.1	640	256	15
Mtb9	LAM1—SIT20	MDR	*inhA* C-15T/S94A	*rpoB* D516V	3	20	256	15
Mtb10	T1—SIT53	MDR	*katG* S315N	*rpoB* H526Y	3	20	256	15
Mtb11	T1—SIT53	MDR	*katG* S315T	*rpoB* S531L	10	640	256	15
Mtb12	T2—Orphan	MDR	*katG* S315T	*rpoB* S531L	10	160	128	15
Mtb13	LAM1—SIT20	MDR	*inhA* C-15T/S94A	*rpoB* S531L	3	320	256	15
Mtb14	Beijing—SIT1	MDR	*katG* S315T	*rpoB* S531L	20	320	256	15
Mtb15	LAM1—SIT20	XDR	*inhA* C-15T/S94A	*rpoB* S531L	20	80	128	15
Mtb16	LAM4—SIT1106	XDR	*inhA* C-15T/I194T	*rpoB* S531L	3	320	256	15
Mtb17	LAM4—SIT1106	XDR	*inhA* C-15T/I194T	*rpoB* S531L	3	320	256	15

Spoligotype SIT/clades were defined according to the international spoligotype database SITVITWEB rules. “Unknown” designates patterns with signatures that do not belong to any of the major lineages defined in the SITVITWEB database. The lowest concentration of antibiotic tested corresponded to the critical concentration of each drug. DST, drug susceptibility testing; MIC, minimum inhibitory concentration; INH, isoniazid; LAM, Latin American–Mediterranean; MDR, multidrug resistant; RIF, rifampicin; SIT, spoligotype international type; XDR, extensively drug resistant; VP, verapamil; TZ, thioridazine; Suscep., susceptible; R, resistant; wt, wild-type sequence; STD, antibiotic susceptibility testing.

**Table 2:** Characterization of the *M. tuberculosis* strains according to their efflux capacity.

Strains	Efflux Activity				
	Span_EtBr_	RFF_VP_	RFF_TZ_	K	t_efflux50%_
**Suscep.**					
**H37Rv**	26.10	1.57 ± 0.02	0.77 ± 0.01	0.09 ± 0.008	7.94 ± 0.75
**Mtb#1**	29.42	1.04 ± 0.03	0.13 ± 0.02	0.09 ± 0.086	8.06 ± 0.63
**Mtb#2**	28.51	2.07 ± 0.09	1.18 ± 0.03	0.09 ± 0.003	7.77 ± 0.30
**INH^R^**					
**Mtb#3**	14.07	0.59 ± 0.02	0.90 ± 0.04	0.06 ± 0.008	11.98 ± 1.81
**Mtb#4**	13.50	1.21 ± 0.02	0.13 ± 0.05	0.08 ± 0.004	8.95 ± 0.48
**RIF^R^**					
**Mtb#5**	12.13	1.00 ± 0.03	0.41 ± 0.01	0.07 ± 0.009	8.55 ± 0.47
**Mtb#6**	13.50	2.31 ± 0.02	1.11 ± 0.01	0.12 ± 0.004	5.95 ± 0.21
**Mtb#7**	10.46	1.26 ± 0.06	0.13 ± 0.05	0.10 ± 0.0004	6.67 ± 0.02
**Mtb#8**	13.16	1.16 ± 0.03	0.28 ± 0.01	0.14 ± 0.004	4.39 ± 0.41
**MDR**					
**Mtb#9**	22.39	1.62 ± 0.09	0.79 ± 0.04	0.11 ± 0.0006	6.49 ± 0.04
**Mtb#11**	7.45	1.31 ± 0.36	0.39 ± 0.06	0.12 ± 0.0057	5.90 ± 0.42
**Mtb#12**	8.86	0.71 ± 0.00	0.30 ± 0.09	0.05 ± 0.0011	14.06 ± 0.46
**Mtb#13**	11.74	2.18 ± 0.01	0.98 ± 0.02	0.10 ± 0.0095	7.25 ± 0.72
**Mtb#14**	12.19	1.21 ± 0.15	0.89 ± 0.06	0.09 ± 0.0036	8.08 ± 0.33
**XDR**					
**Mtb#16**	13.56	1.15 ± 0.17	1.05 ± 0.01	0.10 ± 0.007	6.89 ± 0.69
**Mtb#17**	14.80	1.78 ± 0.22	0.91 ± 0.23	0.14 ± 0.022	4.99 ± 0.78

The effect of the efflux inhibitors on the accumulation of ethidium bromide was interpreted as follows: relative final fluorescence (RFF) indexes above zero indicated that cells accumulate more ethidium bromide under the condition used than those of the control (non-treated bacterial cells). RFF values above 1 indicate enhanced accumulation of ethidium bromide in the presence of the efflux inhibitors. Each assay was performed in triplicate and the results presented correspond to the average of three independent assays plus standard deviation (±SD) [19,32]. Ethidium bromide was used at 0.125 µg/mL for Mtb#2, Mtb#5, Mtb#9; 0.25 µg/mL for H37Rv, Mtb#1, Mtb#4, Mtb#6, Mtb#7, Mtb#13, Mtb#14, Mtb#16, Mtb#17; and 0.5 µg/mL for Mtb#3, Mtb#8, Mtb#11, Mtb#12. Strains Mtb#10 and Mtb#15 were not evaluated due to poor growth under the conditions required for these assays. The inhibitors were tested at 1/2 or 1/4 MIC (see Material and Methods for details). EtBr, ethidium bromide; INH, isoniazid; K, efflux rate constant; MDR, multidrug resistant; R, resistant; RIF, rifampicin; Suscep., susceptible; TZ, thioridazine; VP, verapamil; XDR, extensively drug resistant.

**Table 3:** Synergistic effect between the efflux inhibitors and the antituberculosis drugs against the *M. tuberculosis* strains determined by qDST.

Strain	MIC (µg/mL)									MIC (µg/mL)						
	INH	INH + VP				INH + TZ				RIF	RIF + VP			RIF + TZ		
		0.1	1	3	10	0.1	1	3	10		1	4	20	1	4	20
**Suscep.**																
H37Rv	0.1	S	-	-	-	-	-	-	-	1	S	-	-	-	-	-
Mtb1	0.1	S	-	-	-	-	-	-	-	1	S	-	-	-	-	-
Mtb2	0.1	S	-	-	-	-	-	-	-	1	S	-	-	-	-	-
**INH^R^**																
Mtb3	10	R	R	R	S	R	R	R	S	1	-	-	-	-	-	-
Mtb4	1	R	S	S	S	R	S	S	S	1	-	-	-	-	-	-
**RIF^R^**																
Mtb5	0.1	-	-	-	-	-	-	-	-	340	R	R	R	R	R	R
Mtb6	0.1	-	-	-	-	-	-	-	-	320	R	R	R	R	R	R
Mtb7	0.1	-	-	-	-	-	-	-	-	320	R	R	R	R	R	R
Mtb8	0.1	-	-	-	-	-	-	-	-	640	R	R	R	R	R	R
**MDR**																
Mtb9	3	R	**S**	S	-	R	**S**	S	-	20	R	**S**	S	R	**S**	S
Mtb10	3	R	R	S	-	R	R	S	-	20	**S**	**S**	S	**S**	**S**	S
Mtb11	10	R	R	**S**	S	R	R	R	S	640	R	R	R	R	R	R
Mtb12	10	R	R	**S**	S	R	R	**S**	S	160	R	R	**S**	R	R	R
Mtb13	3	R	**S**	S	-	R	**S**	S	-	320	R	R	**S**	R	R	R
Mtb14	20	R	R	R	**S**	R	R	**S**	**S**	320	R	R	**S**	R	R	R
**XDR**																
Mtb15	20	R	**S**	**S**	**S**	R	R	R	R	80	R	R	**S**	R	R	**S**
Mtb16	3	R	**S**	S	-	R	**S**	S	-	320	R	R	**S**	R	R	R
Mtb17	3	R	**S**	S	-	R	R	S	-	320	R	R	**S**	R	R	R

INH, isoniazid; MDR, multidrug resistant; RIF, rifampicin; XDR, extensively drug resistant; VP, verapamil; TZ, thioridazine; suscep or S, susceptible; R, resistant; MIC, minimum inhibitory concentration. The lowest concentration of antibiotic tested corresponded to the critical concentration of each drug; the highest concentration tested was the one that defines high-level resistance according to the qDST procedure (see Material and Methods for details). Highlighted in red-bold type letter are the changes in the susceptibility (R to S) of the strain to the respective antibiotic at the concentration tested in the presence of the efflux inhibitor. -, not tested.

## Abbreviations

The following abbreviations are used in this manuscript:

AST	antibiotic susceptibility testing
d	days
EtBr	ethidium bromide
h	hours
INH	isoniazid
K	efflux rate constant
LAM	Latin American-Mediterranean
MDR	multidrug resistant
qDST	quantitative drug susceptibility testing
R	resistant
RIF	rifampicin
Suscep/S	susceptible
SIT	spoligotype international type
TB	tuberculosis
TTD	time to detection
TZ	thioridazine
VP	verapamil
wt	wild-type sequence
XDR	extensively drug resistant.
